# Impact of baseline ECG characteristics on changes in cardiac biomarkers and echocardiographic metrices after acute myocardial infarction treated with Empagliflozin

**DOI:** 10.1038/s41598-024-64175-5

**Published:** 2024-07-02

**Authors:** Martin Benedikt, Faisal Aziz, Thomas Fröschl, Christoph Strohhofer, Ewald Kolesnik, Norbert Tripolt, Peter Pferschy, Markus Wallner, Heiko Bugger, Andreas Zirlik, Daniel Scherr, Harald Sourij, Dirk von Lewinski

**Affiliations:** 1 Division of Cardiology, Department of Internal Medicine, Medical University of Graz, Auenbruggerplatz 15, Graz, Austria; 2 Division of Endocrinology and Diabetology, Department of Internal Medicine, Medical University of Graz, Auenbruggerplatz 15, Graz, Austria; 3 Interdisciplinary Metabolic Medicine Trials Unit, Medical University of Graz, Graz, Austria

**Keywords:** Empagliflozin, Myocardial infarction, Electrocardiogram, Heart failure, Electric conduction, Cardiology, Medical research

## Abstract

The EMMY trial was a multicentre, investigator-initiated, placebo-controlled, double-blind trial, which enrolled 476 patients immediately following AMI and the first study demonstrating a significant reduction in NT-proBNP-levels as well as significant improvements in cardiac structure and function in patients after acute myocardial infarction treated with empagliflozin vs. placebo. However, hardly any data are available investigating the prognostic role of baseline electrocardiogram metrics in SGLT2-inhibitor-treated patients. This post-hoc analysis investigated the association of baseline ECG metrics collected in one centre of the trial (181 patients) with changes in structural and functional cardiac parameters as well as cardiac biomarkers in response to Empagliflozin treatment. A total of 181 patients (146 men; mean age 58 ± 14 years) were included. Median PQ-interval was 156 (IQR 144–174) milliseconds (ms), QRS width 92 (84–98) ms, QTc interval 453 (428–478) ms, Q-wave duration 45 (40–60) ms, Q-wave amplitude 0.40 (0.30–0.70) millivolt (mV), and heart rate was 71 (64–85) bpm. For functional cardiac parameters (LVEF and E/eʹ) of the entire cohort, a greater decrease of E/eʹ from baseline to week 26 was observed in shorter QRS width (P = 0.005).Structural cardiac endpoints were only found to have a significant positive correlation between LVEDD and Q wave duration (P = 0.037). Higher heart rate was significantly correlated with better response in LVEF (P = 0.001), E/eʹ (P = 0.021), and NT-proBNP (P = 0.005). Empagliflozin-treatment showed no interaction with the results. Baseline ECG characteristics post AMI are neither predictive for beneficial NTproBNP effects of Empagliflozin post AMI, nor for functional or structural changes within 26 weeks post AMI.

## Introduction

Sodium-glucose co-transporter2-inhibitors (SGLT2-i) have been shown to significantly reduce the composite endpoint of cardiovascular death and hospitalisation for heart failure in patients with chronic heart failure independent of the left-ventricular ejection fraction (LV-EF) or the presence of diabetes^1–5^. Based on these findings, SGLT2-i received a class IA recommendation from the AHA/ACC/HFSA and ESC guidelines for the treatment of patients with heart failure with reduced ejection fraction (HFrEF)^6,7^ as well as for heart failure with mid-range and preserved ejection fraction (HFmrEF and HFpEF)^7,6^. Furthermore, pleiotropic beneficial effects on renal outcomes reducing the composite endpoint for progression of kidney disease were demonstrated for Empagliflozin^8^, Canagliflozin^9^, and Dapagliflozin^10^.

The recently published “Empagliflozin in acute myocardial infarction” (EMMY) trial was the first clinical trial highlighting a significant reduction in N-terminal pro-hormone of brain natriuretic peptide (NTproBNP) levels as well as structural (left ventricular end-diastolic and end-systolic diameter [LVEDD, LVESD]) and functional cardiac parameters (LVEF, E/eʹ) in patients with and without diabetes suffering from acute myocardial infraction (AMI), receiving Empagliflozin in addition to guideline recommended post-MI treatment^11^.

The electrocardiogram (ECG) plays an important role in the initial diagnostic work-up of acute myocardial infarction^12,13^ and can be used as a prognostic tool for patients experiencing an acute myocardial infarction^14^. Katragadda et al. reported a significantly poorer outcome in the presence of reciprocal ECG-changes following ST-segment elevating myocardial infarction (STEMI) and identified lower QRS amplitudes as predictor of infarct size^15^. In patients with diabetes, the use of SGLT2-i’s appeared to be well-tolerated in terms of electrophysiological safety, showing no differences in PR interval, QT interval, ST-T changes, QRS width, and cardiovascular mortality compared to non-SGLT2-i users^16^. Another trial reported a significant increase in QRS duration among diabetes patients with heart failure and decrease in heart rate for both 10 and 25 mg of Empagliflozin compared to placebo^17^. Additionally, in patients suffering from heart failure, a significant increase in QRS duration was identified^17^, but reciprocally showed positive changes in ventricular repolarisation markers in HFrEF^18^. Further, a significant shortening of the P-wave duration was observed for Empagliflozin, potentially reflecting an improvement in left atrial volumes and left ventricular diastolic function^19^.

Nevertheless, clinical data on beneficial effects of SGLT2-inhibition in patients suffering from AMI with specific baseline electrocardiographic metrics are scarce.

This post-hoc analysis investigated baseline ECG metrices and their potential relation with structural and functional cardiac parameters as well as cardiac biomarkers aiming to identify patients that may particularly benefit from treatment with an SGLT2i following AMI.

## Materials and methods

### Study design

This is a post-hoc analysis of the recently published EMMY trial.

EMMY was a 1:1 randomized, multicentre, investigator-initiated, double-blind, and placebo-controlled trial that investigated the potential effects of the SGLT2-i Empagliflozin 10 mg daily on structural (LVEDD, LVESD) as well as functional (LV-ejection fraction, E/Eʹ) cardiac parameters and cardiac biomarkers (Troponin T, NTproBNP) in patients after AMI^20,21^. Specific ECG parameters were measured one day post AMI in study participants at the Medical University of Graz. The aim of the sub-analysis was to investigate potential association of baseline ECG metrics with changes in structural and functional cardiac parameters as well as cardiac biomarkers in response to empagliflozin treatment.

### Ethical aspects

The EMMY trial was registered on ClinicalTrials.gov (NCT03087773) and approved by the relevant regulatory authorities and the Ethics Committee of the Medical University of Graz, Austria (EK 29–179 ex 16/17; EudraCT 2016-004591-22). The EMMY trial was conducted in conformity with the 1964 declaration of Helsinki and all subsequent revisions were carried out in accordance with the guidelines laid down by the International Conference on Harmonization for Good Clinical Practice (ICH GCP E6 guidelines).

### Study variables

Exploratory variables were electrocardiographic parameters of 12-lead ECG recordings 1 day post AMI (P-wave duration and amplitude, QRS width and amplitude, QTc interval, PR interval, cardiac axis, Sokolow–Lyon Index, ST-segment elevation and depression, T-wave inversions, heart rate, Q-wave duration and amplitude) defined as mean duration (milliseconds, ms) and mean amplitude (millivolt, mV). Blood samples were collected and centrally analysed from all study patients at baseline and after 6 and 26 weeks at the clinical institute for medical and chemical laboratory diagnostics (CIMCL) of the Medical University of Graz.

Outcome variables in this post-hoc analysis include NTproBNP, LVEF, E/eʹ, Troponin T, LVESD, LVEDD, LVESV as well as LVEDV.

### Statistical analysis

#### Baseline characteristics

A complete case analysis of the metrics of the Graz Cohort in the EMMY trial with available ECG at baseline was performed. Baseline measurements of clinical characteristics and ECG metrics were summarized as median with interquartile range (IQR) for continuous variables and frequencies with percentages (%) for categorical variables. Baseline measurements of clinical characteristics and ECG metrics were compared with treatment group using Chi-square or Fischer Exact tests for categorical variables and Wilcoxon rank-sum test for continuous variables. P values < 0.05 were considered statistically significant in all performed tests.

#### Association analysis

A linear mixed effect model (LMEM) was fitted to analyze the association of each ECG metric with the percentage change over time for the primary and secondary endpoints of the EMMY trial. The interaction of each ECG metric with empagliflozin treatment was analyzed for each endpoint. The continuous ECG metrics were also transformed into tertiles to investigate the magnitude of change in endpoints with respect to the strata of these metrices (Supplementary Table 2). The results of continuous ECG metrices were reported for tertiles only in the main results. All statistical analyses were conducted in the Stata software version 18.0 (https://www.stata.com). P values < 0.05 were considered statistically significant in all performed tests.

## Results

### Trial population

**Figure 1:**
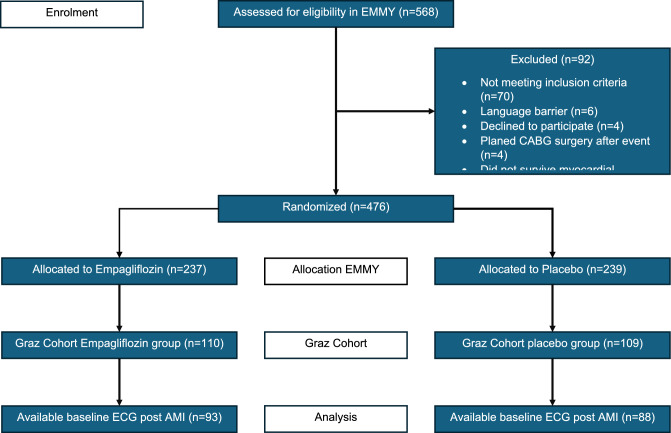
Consort diagram of EMMY and the ECG sub-analysis.

A total of 181 patients enrolled at the study centre at the Medical University of Graz were included in this post-hoc analysis (Fig. 1). Baseline characteristics were distributed equally in the two groups and were comparable to the total EMMY cohort. (Table 1, Supplementary Table 1). The mean age was 58 ± 14 years, 80.7% were male and a mean body mass index (BMI) of 27.7 ± 5.1 kg/m^2^. Cardiovascular risk factors were also balanced between the groups and showed that 13.3% had type-2 diabetes mellitus (T2DM), 69.6% were active or previous smokers, 9.4% had dyslipidaemia, and 34.4% had arterial hypertension. The mean systolic blood pressure was 125 ± 11 mmHg and diastolic blood pressure was 77 ± 8 mmHg. Past medical history of coronary artery disease (CAD) was reported in 6% of patients, history of coronary artery bypass grafting (CABG) in 2.2%, history of stroke in 1.7%, history of carcinoma in 3.9%, and history of depression in 4.4% of patients. Regarding the state of CAD, 42.5% of patients had a 1-vessel disease, 35.9% had a 2-vessel disease, and 21.5% had a 3-vessel disease.

**Table 1:** Baseline characteristics of study participants with available ECG data (N = 181).

Variables	All	Empagliflozin	Placebo	P-value
	N = 181	N = 93	N = 88	
Age (years), median (IQR)	58 (52–66)	57 (52–65)	58 (52–68)	0.621
Gender, n (%)				
Male	146 (80.66)	79 (84.95)	67 (76.14)	0.134
Female	35 (19.34)	14 (15.05)	21 (23.86)	
Body mass index (kg/m^2^), median (IQR)	28 (25–30)	28 (25–31)	28 (25–30)	0.956
Systolic blood pressure (mmHg), median (IQR)	125 (118–129)	126 (117–129)	125 (118–130)	0.771
Diastolic blood pressure (mmHg), median (IQR)	77 (73–81)	76 (73–80)	77 (73–81)	0.453
Smoking (active or former), n (%)	126 (69.61)	65 (69.89)	61 (69.32)	0.933
Type 2 diabetes, n (%)	24 (13.26)	11 (11.83)	13 (14.77)	0.559
Hypertension, n (%)	62 (34.25)	30 (32.26)	32 (36.36)	0.561
Hyperlipidemia, n (%)	17 (9.39)	9 (9.68)	8 (9.09)	0.892
Coronary artery disease, n (%)	12 (6.63)	8 (8.60)	4 (4.55)	0.273
History of CABG, n (%)	2 (1.10)	1 (1.08)	1 (1.14)	0.969
History of stroke, n (%)	3 (1.66)	2 (2.15)	1 (1.14)	0.593
Depression, n (%)	7 (3.87)	1 (1.08)	6 (6.82)	0.045
History of carcinoma, n (%)	8 (4.42)	5 (5.38)	3 (3.41)	0.520
Coronary artery angiography vessel status, n (%)				
1-vessel disease	77 (42.54)	31 (33.33)	46 (52.27)	0.028
2-vessel disease	65 (35.91)	37 (39.78)	28 (31.82)	
3-vessel disease	39 (21.55)	25 (26.88)	14 (15.91)	
Treatment				
ACE-1/ARB, n (%)	179 (100.00)	92 (100.00)	87 (100.00)	–
ARNI, n (%)	4 (2.21)	1 (1.08)	3 (3.41)	0.286
Beta-blocker, n (%)	177 (97.79)	91 (97.85)	86 (97.73)	0.955
MRA, n (%)	101 (55.80)	52 (55.91)	49 (55.68)	0.975
Loop diuretic, n (%)	16 (8.84)	11 (11.83)	5 (5.68)	0.145
Statin, n (%)	181 (100.0)	93 (100.0)	88 (100.0)	–
Ezetimibe, n (%)	3 (1.66)	3 (3.23)	0 (0.00)	0.145
Calcium channel blocker, n (%)	8 (4.42)	2 (2.15)	6 (6.82)	0.127
Platelet aggregation inhibitor, n (%)	181 (100)	93 (100)	88 (100.00)	–
Anticoagulation drugs, n (%)	15 (8.29)	7 (7.53)	8 (9.09)	0.703
Metformin, n (%)	14 (7.73)	6 (6.45)	8 (9.09)	0.506
DPP-4 inhibitor, n (%)	8 (4.42)	4 (4.30)	4 (4.55)	0.936
Sulfonylurea, n (%)	1 (0.55)	1 (1.08)	0 (0.00)	0.329
GLP1-RA, n (%)	2 (1.10)	1 (1.08)	1 (1.14)	0.969
Insulin, n (%)	6 (3.31)	2 (2.15)	4 (4.55)	0.368
Laboratory parameters				
HbA1c (%), median (IQR)	5.60 (5.40–6.00)	5.60 (5.40–6.00)	5.70 (5.40–6.00)	0.523
eGFR (ml/min), median (IQR)	92.68 (78.97–100.16)	92.79 (78.88–99.13)	91.87 (79.31–102.24)	0.758
Haemoglobin (g/dL), median (IQR)	14.00 (13.10–15.00)	14.00 (13.10–14.80)	14.10 (13.10–15.10)	0.461
Creatine kinase (U/L), median (IQR)	1623 (1156–2289)	1491 (1099–2209)	1715 (1219–2347)	0.313
Troponin T (µg/L), median (IQR)	3185 (2099–5117)	3460 (2253–5285)	3070 (2099–5099)	0.479
Total cholesterol (mg/dL), median (IQR)	184 (157–223)	182 (157–223)	187 (158–223)	0.467
LDL-cholesterol (mg/dL), median (IQR)	117 (89–145)	110 (90–144)	121 (87–150)	0.484
HDL-cholesterol (mg/dL), median (IQR)	42 (36–53)	43 (36–52)	42 (36–55)	0.795
Aspartate aminotransferase (U/L), median (IQR)	239 (171–332)	223 (175–332)	255 (142–332)	0.729
Alanine aminotransferase (U/L), median (IQR)	48 (36–74)	49 (34–75)	48 (36–72)	0.640
Gamma glutamyltransferase (U/L), median (IQR)	32 (20–53)	33 (21–57)	31 (19–51)	0.263
Outcomes				
NT-proBNP (pg/m), median (IQR)	1421 (860–2246)	1143 (850–2070)	1650 (950–2539)	0.058
LVEF (%), median (IQR)	47 (43–52)	48 (44–52)	47 (42–52)	0.708
LVEDD (mm), median (IQR)	50 (46–53)	50 (47–53)	49 (46–53)	0.665
LVESD (mm), median (IQR)	37 (33–41)	37 (33–41)	37 (33–41)	0.767
LVEDV (mL), median (IQR)	125 (104–141)	127 (107–143)	123 (102–141)	0.542
LVESV (mL), median (IQR)	66 (52–78)	66 (52–78)	66 (51–78)	0.935
E/eʹ	8.85 (7.47–10.58)	8.84 (7.39–10.56)	8.85 (7.54–11.01)	0.401

Median (IQR): p-value from Wilcoxon rank-sum tests.

Frequency (%): p-value from Chi-square tests.

*CABG* coronary artery bypass graft, *ACE-I* angiotensin-converting enzyme inhibitor, *ARNI* angiotensin-receptor neprilysin inhibitor, *ARB* angiotensin receptor blocker, *MRA* mineralocorticoid receptor antagonist, *DPP-4* dipeptidyl peptidase inhibitor 4, *GLP1-RA* glucagon-like peptide 1 receptor agonist, *eGFR* estimated glomerular filtration rate, *LDL* low-density lipoprotein, *HDL* high-density lipoprotein, *NT-proBNP* N-terminal prohormone of brain natriuretic peptide, *LVEF* left ventricular ejection fraction, *LVEDD* left ventricular enddiastolic diameter, *LVESD* left ventricular endsystolic diameter, *LVEDV* left ventricular enddiastolic volume, *LVESV* left ventricular endsystolic volume, *SD* standard deviation, *IQR* interquartile range.

The median (IQR) level of NTproBNP was 1421 (860–2246) pg/ml, eGFR 92 (78–100) ml/min, creatinine kinase 1623 (1156–2289) U/l, and troponin was 3185 (2099–5117) µg/l.

In functional cardiac parameters, the median (IQR) LVEF was 47 (43–52) % and E/eʹ was 9 (7–11). For structural cardiac parameters, the median LVEDD was 50 (46–53) mm, LVESD was 37 (33–41) mm, LVESV was 66 (52–78) ml, and LVEDV was 125 (104–141) ml (Table 1).

### Baseline ECG parameters

The descriptive statistics of baseline ECG parameters are reported in Table 2. The median (IQR) PQ-interval was 156 (144–174) ms, QRS width was 92 (84–98) ms, QRS amplitude was 1.50 (1.20–1.90) mV, QTc was 453 (428–470) ms, P-wave duration was 100 (80–100) ms, and P-wave amplitude was 0.10 (0.10–0.10) mV. The Sokolow–Lyon index, as a representative for cardiac hypertrophy, showed a median of 1.40 (1.10–1.90) mV. Heart rate was reported with a median of 71 (64–85) bpm in all participants.

**Table 2:** ECG parameters, overall and by treatment.

	All	Empagliflozin	Placebo	P-value
N	181 (100.0%)	93 (51.4%)	88 (48.6%)	
PQ interval (ms)	156.00 (144.00–174.00)	156.00 (144.00–174.00)	156.00 (144.00–172.00)	0.554
QRS amplitude (mV)	1.50 (1.20–1.90)	1.50 (1.20–1.80)	1.60 (1.20–1.95)	0.108
QRS width (ms)	92.00 (84.00–98.00)	90.00 (86.00–100.00)	94.00 (83.00–98.00)	0.506
Sokolow–Lyon index (mV)	1.40 (1.10–1.90)	1.40 (1.10–1.80)	1.50 (1.15–2.00)	0.107
QTc interval (ms)	453.00 (428.00–478.00)	452.00 (426.00–474.00)	454.00 (429.50–482.00)	0.570
P-wave duration (ms)	100.00 (80.00–100.00)	100.00 (80.00–100.00)	90.00 (80.00–100.00)	0.089
P-wave amplitude (mV)	0.10 (0.10–0.10)	0.10 (0.10–0.10)	0.10 (0.10–0.10)	0.149
Cardiac axis				
Normal axis	70 (38.67%)	35 (37.63%)	35 (39.77%)	0.752
Extreme axis deviation	1 (0.55%)	0 (0.00%)	1 (1.14%)	
Left axis deviation	106 (58.56%)	56 (60.22%)	50 (56.82%)	
Right axis deviation	4 (2.21%)	2 (2.15%)	2 (2.27%)	
ST elevation (mV)	0.10 (0.10–0.20)	0.10 (0.10–0.20)	0.10 (0.10–0.20)	0.571
ST elevation				
No	65 (35.91%)	37 (39.78%)	28 (31.82%)	0.264
Yes	116 (64.09%)	56 (60.22%)	60 (68.18%)	
ST depression				
No	132 (72.93%)	69 (74.19%)	63 (71.59%)	0.694
Yes	49 (27.07%)	24 (25.81%)	25 (28.41%)	
T-inversion				
No	42 (23.20%)	21 (22.58%)	21 (23.86%)	0.838
Yes	139 (76.80%)	72 (77.42%)	67 (76.14%)	
Ischemic changes				
No	171 (94.48%)	87 (93.55%)	84 (95.45%)	0.575
Yes	10 (5.52%)	6 (6.45%)	4 (4.55%)	
MI type				
NSTEMI	22 (12.36%)	12 (13.33%)	10 (11.36%)	0.690
STEMI	156 (87.64%)	78 (86.67%)	78 (88.64%)	
Heart rate (bpm)	71.00 (64.00–85.00)	69.00 (62.00–85.00)	71.50 (64.50–84.00)	0.877
Q-wave				
No	91 (50.84%)	46 (50.00%)	45 (51.72%)	0.818
Yes	88 (49.16%)	46 (50.00%)	42 (48.28%)	
Q-wave duration (ms)	45.00 (40.00–60.00)	40.00 (40.00–60.00)	60.00 (40.00–60.00)	0.280
Q-wave duration				
< 40 ms	13 (14.44%)	8 (17.02%)	5 (11.63%)	0.467
≥ 40 ms	77 (85.56%)	39 (82.98%)	38 (88.37%)	
Q-wave amplitude (mV)	0.40 (0.30–0.70)	0.40 (0.30–0.70)	0.50 (0.30–0.80)	0.285

*ms* milliseconds, *mV* millivolt, *STEMI* ST-segment elevating myocardial infarction, *NSTEMI* non-ST-segment elevating myocardial infarction, *bpm* beats per minute.

The electrical axis analysis revealed that 58.6% had left axis deviation, 38.7% normal axis, 2.2% right axis deviation, and 0.55% had extreme axis deviation.

Pathological Q-waves were identified in 49.2% of patients, whereas 85.6% showed a relevant duration of ≥ 40 ms. The median Q-wave duration was 45 (40–65) ms and Q-wave amplitude was 0.40 (0.30–0.70) mV.

The majority of patients (87.6%) experienced ST-elevation myocardial infarction (STEMIs). The ST-elevation was still present in 64.1% of patients 1-day after AMI with a median of 0.10 (0.10–0.20) mV, while ST-depression was present in 27.1% of patients. Further, T-inversions were identified in 76.8% of patients following AMI (Table 2).

### Association of ECG metrics with cardiac outcomes

No interaction between baseline ECG parameters, treatment groups and EMMY outcomes could be detected in the cohort analysed (Table 3). Therefore, both groups (Empagliflozin and placebo) were merged to analyse the association of baseline ECG metrices with changes in cardiac outcomes regardless of SGLT2-i treatment.

**Table 3:** Interaction analysis of ECG parameters and treatment groups with EMMY outcomes.

	Interaction	95% CI		P-interaction
NT-proBNP				
Cardiac axis × treatment				
Left axis deviation	0.17	− 0.32	0.66	0.497
Right axis deviation	− 0.82	− 2.44	0.81	0.323
ST-elevation × treatment	0.23	− 0.27	0.73	0.374
ST-depression1 × treatment	0.05	− 0.49	0.59	0.851
T-inversion × treatment	0.08	− 0.48	0.65	0.778
Ischemic changes × treatment	− 0.40	− 1.48	0.67	0.465
MI type × treatment	0.47	− 0.26	1.21	0.207
Q wave × treatment	0.26	− 0.22	0.75	0.288
Q wave duration × treatment	0.90	− 0.019	0.001	0.051
PQ-interval × treatment	− 0.008	− 0.02	0.00	0.086
QRS-amplitude × treatment	− 0.19	− 0.63	0.26	0.403
QRS-width × treatment	0.009	− 0.01	0.03	0.343
Sokolow lyon index × treatment	− 0.15	− 0.53	0.24	0.453
QTc-interval × treatment	− 0.004	− 0.01	0.00	0.231
P-wave amplitude × treatment				
0.1	0.22	− 0.63	1.08	0.607
0.15	0.28	− 0.79	1.35	0.608
0.2	0.06	− 1.15	1.26	0.925
Heart rate × treatment	− 0.01	− 0.02	0.01	0.392
LVEF				
Cardiac axis × treatment				
Left axis deviation	− 0.02	− 0.10	0.07	0.664
Right axis deviation	− 0.05	− 0.32	0.23	0.743
ST-elevation × treatment	0.02	− 0.07	0.10	0.700
ST-depression1 × treatment	− 0.06	− 0.15	0.03	0.165
T-inversion × treatment	− 0.02	− 0.12	0.07	0.640
Ischemic changes × treatment	− 0.12	− 0.30	0.06	0.185
MI type × treatment	− 0.05	− 0.18	0.07	0.424
Q wave × treatment	− 0.03	− 0.12	0.05	0.406
Q wave duration × treatment	− 0.05	− 0.21	0.11	0.545
PQ-interval × treatment	0.00	− 0.00	0.00	0.286
QRS-amplitude × treatment	0.04	− 0.04	0.11	0.306
QRS-width × treatment	− 0.00	− 0.00	0.00	0.349
Sokolow lyon index × treatment	0.06	− 0.00	0.12	0.071
QTc-interval × treatment	0.00	− 0.00	0.00	0.840
P-wave amplitude × treatment				
0.1	− 0.01	− 0.16	0.13	0.875
0.15	0.09	− 0.09	0.28	0.305
0.2	− 0.01	− 0.21	0.20	0.940
Heart rate × treatment	0.001	− 0.007	0.003	0.378
LVESD				
Cardiac axis × treatment				
Left axis deviation	0.05	− 0.04	0.13	0.279
Right axis deviation	0.04	− 0.24	0.31	0.803
ST-elevation × treatment	0.02	− 0.06	0.11	0.614
ST-depression × treatment	0.04	− 0.05	0.13	0.382
T-inversion × treatment	0.04	− 0.06	0.13	0.466
Ischemic changes × treatment	− 0.01	− 0.20	0.17	0.897
MI type × treatment	0.02	− 0.11	0.14	0.785
Q wave × treatment	− 0.001	− 0.08	0.08	0.994
Q wave duration × treatment	0.10	− 0.07	0.27	0.263
PQ-interval × treatment	− 0.001	− 0.001	0.001	0.164
QRS-amplitude × treatment	− 0.03	− 0.11	0.05	0.442
QRS-width × treatment	− 0.00	− 0.00	0.00	0.751
Sokolow lyon index × treatment	− 0.01	− 0.08	0.05	0.658
QTc-interval × treatment	− 0.00	− 0.00	0.00	0.536
P-wave amplitude × treatment				
0.1	0.04	− 0.11	0.18	0.622
0.15	− 0.06	− 0.25	0.12	0.492
0.2	0.14	− 0.06	0.34	0.179
Heart rate × treatment	− 0.001	− 0.003	0.004	0.236
LVESV				
Cardiac axis × treatment				
Left axis deviation	0.14	− 0.04	0.32	0.135
Right axis deviation	0.18	− 0.41	0.78	0.546
ST-elevation × treatment	− 0.07	− 0.25	0.12	0.470
ST-depression × treatment	0.05	− 0.15	0.24	0.651
T-inversion × treatment	0.04	− 0.16	0.25	0.676
Ischemic changes × treatment	0.27	− 0.11	0.66	0.167
MI type × treatment	0.11	− 0.17	0.38	0.438
Q wave × treatment	− 0.001	− 0.18	0.17	0.982
Q wave duration × treatment	0.16	− 0.18	0.50	0.370
PQ-interval × treatment	− 0.001	− 0.01	0.00	0.493
QRS-amplitude × treatment	− 0.10	− 0.26	0.06	0.226
QRS-width × treatment	− 0.001	− 0.01	0.00	0.572
Sokolow lyon index × treatment	− 0.08	− 0.22	0.05	0.231
QTc-interval × treatment	− 0.00	− 0.00	0.00	0.801
P-wave amplitude × treatment				
0.1	0.08	− 0.24	0.40	0.619
0.15	− 0.001	− 0.40	0.39	0.985
0.2	0.22	− 0.23	0.67	0.333
Heart rate × treatment	− 0.006	− 0.01	− 0.001	0.043
LVEDD				
Cardiac axis × treatment				
Left axis deviation	0.03	− 0.03	0.08	0.307
Right axis deviation	0.01	− 0.17	0.18	0.944
ST-elevation × treatment	0.03	− 0.02	0.08	0.285
ST-depression × treatment	0.001	− 0.06	0.06	0.943
T-inversion × treatment	0.02	− 0.04	0.08	0.572
Ischemic changes × treatment	0.03	− 0.08	0.15	0.580
MI type × treatment	0.01	− 0.07	0.09	0.776
Q wave × treatment	− 0.001	− 0.05	0.05	0.919
Q wave duration × treatment	0.08	− 0.03	0.18	0.169
PQ-interval × treatment	− 0.001	− 0.00	0.00	0.619
QRS-amplitude × treatment	− 0.03	− 0.08	0.02	0.181
QRS-width × treatment	− 0.001	− 0.00	0.00	0.927
Sokolow lyon index × treatment	− 0.01	− 0.05	0.03	0.740
QTc-interval × treatment	− 0.001	− 0.001	0.0004	0.432
P-wave amplitude × treatment				
0.1	0.02	− 0.07	0.11	0.635
0.15	− 0.04	− 0.15	0.08	0.534
0.2	0.12	− 0.01	0.25	0.070
Heart rate × treatment	− 0.0003	− 0.002	0.002	0.784
LVEDV				
Cardiac axis × treatment				
Left axis deviation	0.13	− 0.01	0.28	0.067
Right axis deviation	0.15	− 0.32	0.62	0.529
ST-elevation × treatment	− 0.05	− 0.20	0.10	0.498
ST-depression × treatment	0.01	− 0.14	0.17	0.882
T-inversion × treatment	0.04	− 0.12	0.20	0.634
Ischemic changes × treatment	0.13	− 0.18	0.44	0.408
MI type × treatment	0.08	− 0.14	0.30	0.467
Q wave × treatment	− 0.03	− 0.16	0.11	0.697
Q wave duration × treatment	0.12	− 0.15	0.39	0.387
PQ-interval × treatment	− 0.001	− 0.00	0.00	0.702
QRS-amplitude × treatment	− 0.06	− 0.19	0.06	0.320
QRS-width × treatment	− 0.001	− 0.01	0.00	0.349
Sokolow lyon index × treatment	− 0.04	− 0.15	0.07	0.470
QTc-interval × treatment	− 0.001	− 0.00	0.00	0.973
P-wave amplitude × treatment				
0.1	0.08	− 0.17	0.32	0.546
0.15	0.06	− 0.25	0.37	0.722
0.2	0.20	− 0.15	0.55	0.259
Heart rate × treatment	− 0.005	− 0.01	− 0.0001	0.043
E/eʹ				
Cardiac axis × treatment				
Left axis deviation	0.06	− 0.08	0.20	0.402
Right axis deviation	0.16	− 0.31	0.63	0.505
ST-elevation × treatment	− 0.01	− 0.16	0.14	0.890
ST-depression × treatment	0.05	− 0.11	0.21	0.547
T-inversion × treatment	0.02	− 0.14	0.19	0.808
Ischemic changes × treatment	− 0.03	− 0.35	0.29	0.855
MI type × treatment	0.14	− 0.07	0.36	0.197
Q wave × treatment	0.03	− 0.11	0.17	0.653
Q wave duration × treatment	0.24	− 0.04	0.52	0.096
PQ-interval × treatment	− 0.00	− 0.00	0.00	0.221
QRS-amplitude × treatment	− 0.08	− 0.21	0.05	0.255
QRS-width × treatment	0.006	0.0004	0.002	0.034
Sokolow lyon index × treatment	− 0.14	− 0.25	− 0.03	0.014
QTc-interval × treatment	0.0001	− 0.002	0.002	0.939
P-wave amplitude × treatment				
0.1	0.04	− 0.20	0.28	0.751
0.15	0.08	− 0.22	0.38	0.610
0.2	− 0.23	− 0.58	0.11	0.179
Heart rate × treatment	− 0.0004	− 0.005	0.005	0.874
Troponin T				
Cardiac axis × treatment				
Left axis deviation	0.33	0.02	0.63	0.037
Right axis deviation	0.17	− 0.85	1.18	0.746
ST-elevation × treatment	0.09	− 0.22	0.40	0.574
ST-depression × treatment	0.12	− 0.22	0.46	0.495
T-inversion × treatment	0.14	− 0.21	0.49	0.440
Ischemic changes × treatment	− 0.10	− 0.78	0.58	0.781
MI type × treatment	0.49	0.03	0.95	0.036
Q wave × treatment	− 0.07	− 0.37	0.24	0.668
Q wave duration × treatment	0.42	− 0.14	0.99	0.142
PQ-interval × treatment	− 0.004	− 0.011	0.002	0.173
QRS-amplitude × treatment	− 0.03	− 0.31	0.25	0.821
QRS-width × treatment	0.013	0.002	0.024	0.021
Sokolow lyon index × treatment	0.01	− 0.14	0.15	0.943
QTc-interval × treatment	0.001	− 0.00	0.01	0.249
P-wave amplitude × treatment				
0.1	0.36	− 0.18	0.90	0.190
0.15	0.47	− 0.21	1.14	0.178
0.2	0.39	− 0.37	1.15	0.313
Heart rate × treatment	0.002	− 0.01	0.01	0.685

*CI* confidence interval, *LVEF* left ventricular ejection fraction, *LVESD* left ventricular endsystolic diameter, *LVEDD* left ventricular enddiastolic diameter, *LVESV* left ventricular endsystolic volume, *LVEDV* left ventricular enddiastolic volume.

#### Laboratory biomarkers

**Figure 2:**
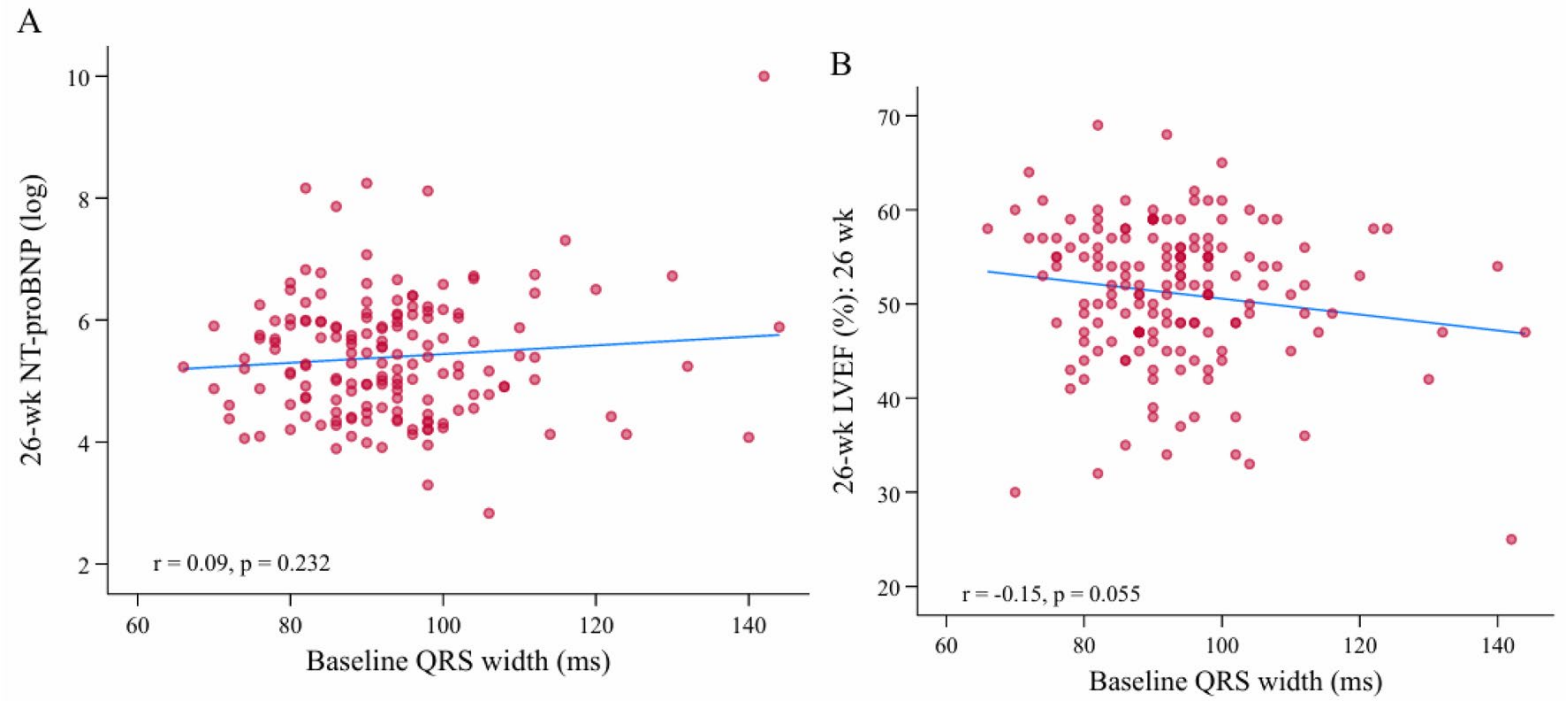
Correlation plots of QRS width (in ms) with NTproBNP and LVEF at week 26.

NTproBNP levels had no significant correlation with ST-elevation (P = 0.897), type of AMI (P = 0.183), the presence of Q-waves (P = 0.252), Q-wave duration (P = 0.44), Q-wave amplitude (P = 0.959), PQ interval (P = 0.878), QRS width (P = 0.32), QTc interval (0.704), and Sokolow–Lyon index (P = 0.055) (Fig. 2, Table 4). The analysis of Troponin T and left ventricular volumes (LVESV and LVEDV) did not reveal any significant associations (Supplementary Tables 3–5).

**Table 4:** Changes in cardiac markers over visits with respect to baseline ECG parameters (N = 181).

ECG parameters	NTproBNP			LVEF			E/eʹ			LVEDD			LVESD		
	% change	P	P_int_	% change	P	P_int_	% change	P	P_int_	% change	P	P_int_	% change	P	P_int_
Cardiac axis deviation															
Extreme	− 79 (− 79, − 79)	0.022	0.419	− 20 (− 20, − 20)	0.435	0.883	− 5 (− 5, − 5)	0.147	0.612	15 (15, 15)	0.093	0.582	7 (7, 7)	0.330	0.555
Left	− 84 (− 90, − 75)			10 (0, 20)			− 16 (− 24, 4)			4 (− 3, 10)			3 (− 3, 11)		
Normal	− 86 (− 92, − 79)			12 (0, 21)			− 5 (− 20, 11)			4 (− 2, 9)			3 (− 7, 11)		
Right	− 61 (− 67, − 49)			4 (2, 15)			− 17 (− 28, 23)			11 (9, 15)			13 (9, 21)		
ST-elevation															
No	− 84 (− 91, − 74)	0.897	0.372	6 (0, 16)	0.277	0.702	− 17 (− 24, 2)	0.095	0.883	2 (− 4, 9)	0.079	0.287	0 (− 9, 11)	0.0244	0.614
Yes	− 84 (− 90, − 75)			12 (0, 21)			− 10 (− 22, 8)			4 (− 2, 11)			5 (− 3, 13)		
ST-depression															
No	− 84 (− 91, − 75)	0.635	0.854	11 (1, 21)	0.184	0.164	− 11 (− 24, 6)	0.911	0.544	4 (− 2, 10)	0.788	0.936	3 (− 5, 11)	0.219	0.379
Yes	− 84 (− 90, − 74)			7 (− 2, 17)			− 12 (− 23, 7)			4 (− 2, 10)			5 (− 3, 14)		
T-inversion															
No	− 80 (− 89, − 70)	0.052	0.779	9 (0, 15)	0.229	0.636	− 16 (− 25, 1)	0.232	0.810	5 (− 2, 10)	0.577	0.568	4 (− 7, 14)	0.956	0.463
Yes	− 85 (− 91, − 75)			11 (0, 21)			− 11 (− 22, 7)			4 (− 2, 10)			3 (− 4, 11)		
Ischemic change															
No	− 85 (− 91, − 75)	0.134	0.458	10 (0, 20)	0.581	0.186	− 10 (− 22, 7)	0.003	0.859	4 (− 2, 10)	0.229	0.575	3 (− 4, 12)	0.461	0.899
Yes	− 78 (− 81, − 71)			4 (0, 15)			− 23 (− 28, − 21)			− 2 (− 8, 8)			0 (− 9, 11)		
MI type															
NSTEMI	− 79 (− 89, − 71)	0.183	0.205	3 (− 2, 19)	0.379	0.425	− 22 (− 28, − 14)	0.018	0.196	9 (− 2, 13)	0.232	0.777	5 (− 7, 14)	0.953	0.786
STEMI	− 85 (− 91, − 76)			11 (0, 20)			− 10 (− 22, 6)			4 (− 2, 9)			3 (− 4, 11)		
Q-wave															
No	− 83 (− 90, − 76)	0.252	0.291	12 (0, 17)	0.666	0.406	− 14 (− 24, 6)	0.258	0.653	4 (− 2, 10)	0.603	0.915	3 (− 3, 13)	0.687	0.992
Yes	− 86 (− 92, − 74)			9 (0, 22)			− 10 (− 21, 6)			4 (− 2, 10)			4 (− 6, 11)		
Q-wave duration															
< 40 ms	− 90 (− 93, − 72)	0.440	0.051	9 (− 2, 23)	0.797	0.547	− 14 (− 21, 10)	0.643	0.096	0 (− 7, 4)	0.037	0.165	3 (− 11, 11)	0.533	0.260
≥ 40 ms	− 84 (− 91, − 74)			10 (0, 21)			− 10 (− 22, 5)			4 (− 1, 10)			4 (− 4, 11)		
P-wave amplitude															
0.05	− 80 (− 93, − 62)	0.333	0.939	8 (0, 14)	0.125	0.456	− 0 (− 13, 6)	0.421	0.221	4 (− 2, 9)	0.814	0.091	7 (− 7, 21)	0.064	0.205
0.10	− 84 (− 90, − 75)			9 (0, 20)			− 12 (− 23, 6)			4 (− 2, 11)			3 (− 3, 11)		
0.15	− 90 (− 93, − 81)			15 (4, 26)			− 22 (− 26, 4)			4 (− 4, 9)			0 (− 12, 10)		
≥ 0.20	− 88 (− 90, − 76)			5 (1, 16)			− 13 (− 20, − 1)			8 (− 4, 11)			12 (2, 21)		
Q-wave amplitude (mV)															
1st tertile	− 87 (− 91, − 73)	0.959	0.042	10 (2, 17)	0.513	0.927	− 12 (− 21, 10)	0.312	0.413	3 (− 2, 6)	0.053	0.656	8 (− 11, 14)	0.958	0.986
2nd tertile	− 84 (− 91, − 75)			6 (0, 21)			− 7 (− 14, 5)			2 (− 2, 10)			3 (− 6, 11)		
3rd tertile	− 86 (− 92, − 70)			13 (1, 23)			− 15 (− 25, 3)			7 (3, 12)			2 (− 3, 9)		
PQ interval															
1st tertile	− 85 (− 90, − 74)	0.878	0.477	6 (0, 17)	0.094	0.808	− 15 (− 24, 0)	0.158	0.897	4 (− 2, 10)	0.699	0.075	3 (− 3, 12)	0.948	0.568
2nd tertile	− 86 (− 91, − 75)			13 (4, 21)			− 8 (− 19, 3)			4 (− 1, 9)			3 (− 4, 11)		
3rd tertile	− 84 (− 91, − 79)			9 (− 2, 20)			− 13 (− 25, 10)			4 (− 2, 11)			5 (− 5, 14)		
QRS amplitude															
1st tertile	− 84 (− 90, − 75)	0.857	0.229	11 (0, 21)	0.528	0.512	− 12 (− 22, 7)	0.979	0.663	5 (− 2, 13)	0.153	0.594	5 (− 3, 14)	0.406	0.674
2nd tertile	− 82 (− 92, − 71)			9 (− 2, 16)			− 14 (− 24, 10)			4 (− 2, 9)			3 (− 4, 12)		
3rd tertile	− 86 (− 90, − 80)			11 (2, 17)			− 7 (− 21, 5)			2 (− 2, 6)			3 (− 9, 10)		
QRS width															
1st tertile	− 83 (− 91, − 76)	0.320	0.254	12 (2, 21)	0.489	0.435	− 13 (− 23, 5)	0.005	0.739	4 (− 2, 11)	0.569	0.123	5 (− 5, 13)	0.244	0.132
2nd tertile	− 86 (− 91, − 79)			11 (0, 16)			− 19 (− 24, − 5)			4 (− 2, 8)			0 (− 3, 8)		
3rd tertile	− 83 (− 90, − 71)			6 (− 2, 20)			1 (− 16, 12)			4 (− 2, 9)			5 (− 3, 15)		
QTc interval															
1st tertile	− 85 (− 90, − 74)	0.704	0.329	7 (0, 16)	0.600	0.916	− 12 (− 27, 5)	0.190	0.803	4 (0, 10)	0.211	0.333	7 (− 3, 17)	0.100	0.633
2nd tertile	− 83 (− 89, − 76)			13 (2, 20)			− 5 (− 18, 7)			4 (− 2, 11)			2 (− 7, 11)		
3rd tertile	− 87 (− 92, − 72)			10 (0, 22)			− 15 (− 23, 4)			2 (− 6, 10)			1 (− 4, 10)		
Sokolow–Lyon index															
1st tertile	− 80 (− 88, − 71)	0.055	0.323	10 (0, 20)	0.769	0.102	− 13 (− 24, 1)	0.232	0.245	6 (− 1, 13)	0.009	0.822	6 (− 3, 14)	0.093	0.756
2nd tertile	− 86 (− 92, − 73)			10 (0, 17)			− 9 (− 22, 12)			4 (− 2, 9)			3 (− 3, 14)		
3rd tertile	− 86 (− 91, − 80)			10 (2, 22)			− 11 (− 22, 4)			2 (− 4, 4)			0 (− 7, 8)		
Heart rate (bpm)															
1st tertile	− 86 (− 91, − 81)	0.005	0.628	4 (− 2, 13)	0.001	0.571	− 12 (− 22, 2)	0.021	0.219	4 (− 4, 6)	0.061	0.035	4 (− 5, 15)	0.363	0.422
2nd tertile	− 86 (− 91, − 79)			14 (6, 22)			− 17 (− 27, 2)			3 (− 2, 9)			3 (− 7, 9)		
3rd tertile	− 79 (− 88, − 70)			12 (− 2, 20)			− 5 (− 20, 12)			8 (− 1, 12)			3 (− 2, 14)		

% change from baseline to 26-week is presented as median with Interquartile range.

*P* P-value for the difference in % change with respect to ECG parameters, *P*_*int*_ P-value for interaction between ECG parameter and treatment, *NTproBNP* B-type natriuretic peptide, *LVEF* left ventricular ejection fraction, *LVEDD* left ventricular end-diastolic diameter, *LVESD* left ventricular end-systolic diameter, *IQR* interquartile range, *ms* milliseconds, *mV* millivolt, *STEMI* ST-segment elevating myocardial infarction, *NSTEMI* non-ST-segment elevating myocardial infarction.

#### Functional cardiac parameters

LVEF as key functional parameter revealed no significant correlation with ST-elevation (P = 0.277), type of AMI (P = 0.379), presence of Q-waves (P = 0.666), Q-wave duration (P = 0.797), Q-wave amplitude (P = 0.513), PQ interval (P = 0.094), QRS width (P = 0.498), QTc interval (P = 0.60), and Sokolow-Lyon index (P = 0.769) (Fig. 2, Table 4).

E/eʹ showed no significant correlation with ST-elevation (P = 0.095), presence of Q-waves (P = 0.258), Q-wave duration (P = 0.643), Q-wave amplitude (P = 0.312), PQ interval (P = 0.158), QTc interval (P = 0.19), and Sokolow–Lyon index (P = 0.232), but significant positive correlations were found for QRS width (P = 0.005) highlighting better response of diastolic function in smaller QRS width (Table 4).

#### Structural cardiac parameters

Structural cardiac parameters were observed to have no significant correlation between LVEDD and ST-elevation (P = 0.079), type of AMI (P = 0.232), presence of Q-waves (P = 0.603), Q-wave amplitude (P = 0.053), PQ interval (P = 0.699), QRS width (P = 0.569), and QTc interval (P = 0.211), but significant correlations were identified for Sokolow–Lyon index (P = 0.0.009) and Q-wave duration (P = 0.037) (Table 4).

Similar results were found for LVESD and type of AMI (P = 0.953), presence of Q-waves (P = 0.687), Q-wave duration (P = 0.533), Q-wave amplitude (P = 0.958), PQ interval (P = 0.948), QRS width (P = 0.244), QTc interval (P = 0.10), and Sokolow-Lyon index (P = 0.769) with significant correlations to ST-elevation (P = 0.0244) (Table 4).

The heart rate was found to have significant positive associations with changes in NTproBNP (P = 0.005), LVEF (P = 0.001), and E/eʹ (P = 0.021) highlighting worse response of functional cardiac parameters as well as lower reduction of NTproBNP in higher heart rate.

## Discussion

The administration of 10 mg Empagliflozin has beneficial cardiac effects including reduction in NTproBNP levels as well as improvements in structural (LVESV and LVEDV) and functional cardiac parameters (LVEF, E/eʹ) compared to placebo after AMI if administered early after PCI^20^. Furthermore, the very early administration of SGLT2-inhibitors appears to have no disadvantages with respect to safety post AMI and was found to be effective in reducing NTproBNP levels and structural as well as functional cardiac parameters^22^.

Early ECG is essential in the initial diagnosis as well as in the follow-up of AMI^12,13^. A prolongation of electrocardiographic parameters such as QRS width or QTc interval is often reported post AMI and was identified to be associated with adverse cardiac outcome^17^. In patients with diabetes, SGLT2-inhibition appeared to be well-tolerated in terms of electrocardiographic changes by showing no significant difference in the duration of PR interval, QT interval and QRS width as well as no relevant changes in ST-T segments compared to non-SGLT2-i users^16^, however data on potential beneficial effect of SGLT2-Inhibition in patients suffering from MI expressed by ECG changes are missing.

Increased QRS width was observed to be often found in patients suffering from heart failure^17^ and is associated with a higher incidence of worsening of HF, sudden death (SD), and cardiovascular death post MI^23^. Hence, these patients appear to be at higher risk for hospitalisation for heart failure and might even more from a treatment with SGLT2-inhibitors. Similarly, a prolongation of the QTc interval in chronic heart failure patients was assessed to be an independent predictor for all-cause mortality, sudden death and progressive heart failure death^24^. Further, in symptomatic ischemic cardiomyopathy and left ventricular systolic dysfunction lethal outcomes following Q-wave infarction were revealed^25^. This sub-analysis reports a prolonged QTc interval as well as Q-wave duration one day post MI, whereas median QRS was normal (Table 2). However, no significant correlations were identified between these parameters and LVEF after 6 and 26 weeks in the overall cohort in our post-hoc analysis. Based on these findings supported by the result of the EMMY trial showing a significant increase in LVEF with Empagliflozin, baseline ECG parameters are not predictive for the better LVEF response to Empagliflozin.

Positive correlations of cardiac diastolic function parameters (LAV, LAVI, E/A) and QTc interval have already been prescribed by Li et al. revealing protective effects of moderate QTc intervals on diastolic function^26^. Further, a significant shortening of P-wave duration was observed for Empagliflozin reflecting an improvement in left atrial volume or conduction^19^. QRS width significantly correlated with E/eʹ, highlighting a better response of diastolic function in patients with a smaller QRS, which could be in line with potential anti-remodelling effects of early applied neurohumoral therapy post AMI. This analysis identified no further significant correlation of baseline ECG parameters and E/eʹ indicating no predictive value of baseline ECG findings.

NTproBNP levels were found to be a predictor in cardiovascular outcome and a strong significant clinical predictor for clinical events when combined with the prolongation of the QTc interval in STEMI patients^27^. Moreover, NTproBNP as well as troponin levels after AMI also offer a good estimation of infarct size^28^ and were associated with worsening of heart failure^29^, whereas troponin levels were significantly associated with QRS duration^30^. This highlights that large infarct size with LV-dysfunction, manifesting with highly elevated troponin and NTproBNP levels, are at higher risk of developing a ventricular conduction disorder with prolongation of the QRS width predicting worse cardiac outcome. Nevertheless, our analysis revealed no significant correlation between baseline ECG findings and NTproBNP as well as Troponin T levels, supporting the notion that baseline ECG characteristics are neither predictive for NTproBNP or troponin changes over time.

SGLT2-inhibitors and Glucagon-like peptide-1 (GLP-1) receptor agonists show blood pressure lowering effects, but only GLP-1 receptor agonists increase heart rate^31^, whereas Empagliflozin did not have a significant impact on heart rate in a previous investigation^17^. Large clinical outcome trials have reported that higher heart rates are associated with worsening of chronic heart failure^32–34^ and an increase in troponin levels in AMI^35^. Supported by the data that Empagliflozin significantly increases systolic as well as diastolic function post MI^20^, and in line with the data from this analysis, no significant increase in heart rate was revealed with SGLT2-inhibitor treatment^17^. However, significant positive correlations of heart rate with LVEF, E/eʹ, and NTproBNP were identified for having a response of functional cardiac parameters as well as the decrease of NTproBNP is worse in higher heart rates that goes in line with adverse cardiac remodelling, highlighting the importance of early initiation of a neurohumoral therapy post AMI including betablockers.

A large meta-analysis reported significant improvement of LVEF, E/eʹ, LVESD, LVEDD, LVM and LAVI in patients treated with SGLT2-inhibitors independent of diabetes status^36^, which is in line with the result of the EMMY trial for AMI^20^. Larger left ventricular volumes were also found to be associated with QRS width as well as Q-waves after anterior MI and was inversely associated with LVEF^37^. The potential for better LV-recovery in HFrEF treated with ARNIs was described to be better in patients with shorter QRS complex^38^. Our analysis did not identify any significant associations of LV-volumes and LV-diameters with baseline QRS width. LVEDD was only found to have a significant positive correlation to Q-wave duration, highlighting that infarct size with myocardial scaring is predictive for adverse cardiac remodeling. However, patients receiving SGLT2-i treatment post AMI were found to have a significant reduction in LV-size in the EMMY trial compared to patients not receiving SGLT2-inhibition^11^ and appear to show positive effects regarding cardiac remodeling independent of baseline electrocardiographic characteristics.

EMMY reported a significant reduction in cardiac biomarkers as well as functional and structural cardiac parameters, however, the trial was not powered for hard clinical endpoints. First evidence of SGLT2-I use post AMI arrived from the “Dapagliflozin in Myocardial Infarction without Diabetes or Heart Failure” (DAPA-MI) trial, which identified significant benefits in improvement of cardiometabolic outcomes but no impact on the composite of cardiovascular death or hospitalization for heart failure compared with placebo was observed^39^. Furthermore, the primary outcome was independent of LVEF. In the recently published outcome trial “Empagliflozin after Acute Myocardial Infarction” (EMPACT-MI) trial Empagliflozin did not significantly lower the risk of hospitalization of heart failure or all-cause death post AMI compared to placebo^40^. Based on the findings of the huge clinical outcome trials and the EMMY trial, SGLT2-i appear to have beneficial effects post MI independent of diabetes status, however, further research in this area must be performed.

## Conclusion

The EMMY trial highlights ameliorating effects of SGLT2-inhibition after AMI by significantly reducing NTproBNP levels and showing significant effects on structural and functional cardiac parameters, however DAPA-MI as well as EMPACT-MI failed to show significant difference concerning hospitalisation from heart failure and all-cause death and thus, further research in this area must be performed.

This post-hoc analysis revealed no differences between the empagliflozin treated patients and the placebo group with respect to correlations of baseline ECG metrics and the primary as well as secondary endpoints. After merging both groups, a conclusive and clinical meaningful pattern of baseline ECG metrics and functional or structural changes within 26 weeks post MI could still not be detected.

The use of empagliflozin after AMI appears to be safe with respect to baseline ECG metrics and patients might therefore benefit from early initiation of an SGLT2-inhibitor regardless of baseline electrocardiographic findings.

## Strengths and study limitations

In this post-hoc analysis, baseline ECGs of only 181 patients (Graz cohort) were available for complete analysis (38% of the entire EMMY cohort), limiting the power of the analysis. A large number of statistical tests were conducted for this analysis, therefore a number of statistical significances must be expected by chance and we did not adjust for multiple testing. Thus, the significant changes described in the analysis need to be interpreted carefully.

This post-hoc analysis only includes baseline ECG measures 1 day post-MI, we did not perform ECGs in the upcoming visits and therefore potential ECG trajectories cannot be identified and analysed in our patients.

P-wave duration could be successfully measured in all included patients, however only 5 patients had a prolonged P-wave durations) and therefore, a correlation analysis between endpoints and P-wave duration (≥ 120 ms and < 120 ms) was not feasible.

Given the small number of patients and lack of follow-up ECGs up to week 26, this sub-analysis offers the first clinical evidence showing baseline ECG-independent changes in response of cardiac biomarkers as well as structural and functional cardiac endpoints in SGLT2-I users and non-SGLT2-I users post AMI, however the results did not differ between Empagliflozin and placebo.

### Supplementary Information

Supplementary Tables.

## Data Availability

The datasets used and/or analysed during the current study available from the corresponding author on reasonable request.
