# Supplementary material for: Impact of baseline ECG characteristics on changes in cardiac biomarkers and echocardiographic metrices after acute myocardial infarction treated with Empagliflozin
Source: Sci Rep. 2024 Jul 2;14:15083. doi: 10.1038/s41598-024-64175-5 (PMC11219811; doi:10.1038/s41598-024-64175-5)
Supplement: Supplementary file 1 — Supplementary Tables. [file 41598_2024_64175_MOESM1_ESM.docx]

**Supplement table 1.** Baseline characteristics in the entire EMMY cohort

| **Characteristics** | **Entire EMMY cohort** | | | |
| --- | --- | --- | --- | --- |
|  | **All** | **Empagliflozin** | **Placebo** | **P-value** |
| All, n (%) | 476 | 237 (49.79) | 239 (50.21) | -- |
| Sex, n (%) |  |  |  |  |
| Male | 392 (82.35) | 195 (82.28) | 197 (82.43) | 0.966 |
| Female | 84 (17.65) | 42 (17.57) | 42 (17.72) |  |
| Age (years), mean ±SD | 57.66 ±9.52 | 57.53 9.03 | 57.78 ±10.01 | 0.774 |
| BMI (kg/m^2^), mean ±SD | 27.98 ±4.48 | 28.23 ±4.22 | 27.72 ±4.72 | 0.213 |
| Diabetes, n (%) | 63 (13.24) | 30 (12.66) | 33 (13.81) | 0.711 |
| Systolic BP (mmHg), mean ±SD | 127.00 ±13.79 | 126.65 ±14.74 | 127.34 ±12.79 | 0.582 |
| Diastolic BP (mmHg), mean ±SD | 80.18 ±8.77 | 80.11 ±8.83 | 80.25 ±8.74 | 0.865 |
| Smoking (active or former), *n* (%) | 341 (71.94) | 171 (72.15) | 170 (71.73) | 0.919 |
| Dyslipidemia, n (%) | 135 (28.36) | 71 (29.96) | 64 (26.78) | 0.442 |
| Hypertension, n (%) | 199 (41.81) | 92 (38.82) | 107 (44.77) | 0.188 |
| CAD, n (%) | 53 (11.53) | 28 (11.81) | 25 (10.46) | 0.639 |
| Stroke, n (%) | 6 (1.26) | 5 (2.11) | 1 (0.42) | 0.121 |
| ACS history, n (%) | 23 (4.83) | 14 (5.91) | 9 (3.77) | 0.276 |
| PAD, n (%) | 8 (1.68) | 5 (2.11) | 3 (1.26) | 0.468 |
| **Laboratory parameters** |  |  |  |  |
| eGFR (mL/min/173m^2^), median (IQR) | 92 (78–102) | 92 (78–101) | 91 (78–102) | 0.883 |
| Creatine kinase (U/L), median (IQR) | 1673 (1202–2456) | 1668 (1136–2532) | 1701 (1254–2404) | 0.71 |
| Troponin T (ng/L), median (IQR) | 3039 (2037–4856) | 3059 (2082–4775) | 3029 (1980–4856) | 0.56 |
| Total cholesterol (mg/dL), mean ±SD | 191.93 ±45.46 | 192.49 ±45.16 | 191.40 ±45.83 | 0.796 |
| LDL-cholesterol, (mg/dL), mean ±SD | 121.69 ±41.25 | 122.06 ±40.00 | 121.33 ±42.53 | 0.851 |
| HDL-cholesterol (mg/dL), median (IQR) | 44 (36–54) | 44 (36–52) | 43 (36–54) | 0.767 |
| LVEF (%), median (IQR) | 48 (43–53) | 48 (43–53) | 49 (43–54) | 0.100 |
| E/e‘, median (IQR) | 8.94 (7.50–10.86) | 8.94 (7.44–10.94) | 8.94 (7.54–10.81) | 0.609 |
| NT-proBNP (pg/mL), median (IQR) | 1294 (757–2246) | 1273 (773–2249) | 1373 (754–2217) | 0.905 |
| **Treatment** |  |  |  |  |
| ACE-I/ARB, n (%) | 459 (97.66) | 228 (97.44) | 231 (97.88) | 0.749 |
| Beta-blocker, n (%) | 457 (96.41) | 223 (94.89) | 234 (97.91) | 0.078 |
| MRA, n (%) | 180 (37.39) | 86 (36.60) | 94 (39.33) | 0.540 |
| Statin, n (%) | 462 (97.47) | 229 (97.45) | 233 (97.49) | 0.976 |
| Ezetimibe, n (%) | 59 (12.45) | 29 (12.34) | 30 (12.55) | 0.944 |
| Platelet inhibitory drugs, n (%) | 476 (100.00) | 237 (100.00) | 239 (100.00) | 1.000 |
| Anticoagulation drugs, n (%) | 37 (7.79) | 16 (6.78) | 21 (8.79) | 0.414 |
| Metformin, n (%) | 41 (8.63) | 21 (8.90) | 20 (8.37) | 0.837 |
| GLP1-RA, n (%) | 4 (0.84) | 2 (0.85) | 2 (0.84) | 0.990 |

*BMI, body mass index; BP, blood pressure; CAD, coronary artery disease; AMI, acute myocardial infarction; PAD, peripheral artery disease; eGFR, estimated glomerular filtration rate; LVEF, left ventricular ejection fraction; NT-proBNP, N-terminal prohormone of brain natriuretic peptide; SD, standard deviation; IQR, interquartile range, LDL, low-density lipoprotein; HDL, high-density lipoprotein; ACE-I, angiotensin-converting enzyme inhibitor; ARB, angiotensin receptor blocker; MRA, mineralocorticoid receptor antagonist; GLP1-RA, glucagon-like peptide 1 receptor agonist*

**Supplement table 2.** Tertiles of ECG metrics

| **ECG metrics** | **1st tertile** | **2nd tertile** | **3rd tertile** |
| --- | --- | --- | --- |
|  | **Min - Max** | **Min - Max** | **Min - Max** |
| Sokolow Index | 0.3 – 1.2 | 1.3 – 1.7 | 1.8 – 4 |
| Q-wave amplitude | 0.1 – 0.3 | 0.35 – 0.6 | 0.7 – 2.5 |
| PQ interval | 102 – 148 | 150 – 166 | 168 – 236 |
| QRS amplitude | 0.6 – 1.3 | 1.4 – 1.8 | 1.9 – 4.4 |
| QRS width | 66 – 88 | 90 – 96 | 98 – 144 |
| QTc interval | 313 – 440 | 441 – 466 | 467 – 582 |
| Heart rate | 48 – 66 | 67 – 80 | 81 – 115 |

*ECG, electrocardiogram; min, minimal; max, maximal*

**Supplement table 3.** Changes in LVESV over visits with respect to baseline ECG parameters (N = 181)

| **LVESV** | **Baseline** | **6-weeks** | **26-weeks** | **% change** | **P-value** | **P-interaction** |
| --- | --- | --- | --- | --- | --- | --- |
|  | **Median (IQR)** | **Median (IQR)** | **Median (IQR)** | **Median (IQR)** |  |  |
| Cardiac axis deviation |  |  |  |  |  |  |
| Extreme axis | 68 (68-68) | 86 (86-86) | 98 (98-98) | 44 (44,44) | 0.245 | 0.307 |
| Left axis | 65 (51-77) | 62 (52-75) | 62 (52-78) | -2 (-16,15) |  |  |
| Normal axis | 68 (52-80) | 64 (52-84) | 62 (52-89) | -1 (-15,14) |  |  |
| Right axis | 62 (48-82) | 69 (64-74) | 74 (74-78) | 20 (-5,55) |  |  |
| ST-elevation |  |  |  |  |  |  |
| No | 65 (52-76) | 64 (55-77) | 62 (53-79) | 1 (-13,20) | 0.269 | 0.470 |
| Yes | 67 (52-78) | 64 (52-78) | 63 (53-80) | -3 (-16,13) |  |  |
| ST-depression |  |  |  |  |  |  |
| No | 66 (52-79) | 63 (54-77) | 62 (54-80) | -1 (-15,17) | 0.795 | 0.646 |
| Yes | 64 (52-74) | 66 (50-82) | 63 (48-78) | -2 (-16,16) |  |  |
| T-inversion |  |  |  |  |  |  |
| No | 65 (51-73) | 64 (52-84) | 62 (51-78) | 2 (-15,28) | 0.342 | 0.672 |
| Yes | 67 (53-79) | 64 (52-77) | 63 (53-80) | -2 (-15,13) |  |  |
| Ischemic change |  |  |  |  |  |  |
| No | 67 (53-78) | 64 (53-80) | 64 (53-80) | -2 (-16,16) | 0.606 | 0.166 |
| Yes | 52 (48-71) | 62 (51-70) | 62 (51-63) | 0 (-4,13) |  |  |
| MI type |  |  |  |  |  |  |
| NSTEMI | 59 (51-75) | 64 (56-74) | 62 (57-74) | -3 (-14,21) | 0.724 | 0.436 |
| STEMI | 67 (52-78) | 64 (52-80) | 62 (52-80) | -1 (-16,15) |  |  |
| Q-wave |  |  |  |  |  |  |
| No | 65 (51-78) | 64 (53-75) | 61 (51-78) | -1 (-18,20) | 0.393 | 0.982 |
| Yes | 67 (55-78) | 65 (52-83) | 65 (53-89) | -1 (-11,15) |  |  |
| Q-wave duration (ms) |  |  |  |  |  |  |
| <40 ms | 57 (51-66) | 56 (41-63) | 54 (51-59) | -6 (-11,4) | 0.516 | 0.369 |
| ≥40 ms | 68 (56-78) | 68 (54-85) | 66 (54-93) | -1 (-13,16) |  |  |
| P-wave amplitude (mV) |  |  |  |  |  |  |
| 0.05 | 72 (65-78) | 69 (55-77) | 64 (53-75) | -13 (-18,13) | 0.221 | 0.723 |
| 0.10 | 64 (52-77) | 64 (53-78) | 62 (53-85) | 0 (-13,20) |  |  |
| 0.15 | 66 (46-88) | 58 (48-80) | 60 (49-76) | -6 (-29,4) |  |  |
| ≥0.20 | 65 (41-82) | 68 (45-76) | 75 (47-80) | 4 (-10,25) |  |  |
| Q-wave amplitude-tertiles |  |  |  |  |  |  |
| Tertile 1 | 63 (53,76) | 62 (55,73) | 58 (51,66) | -3 (-11,2) | 0.308 |  |
| Tertile 2 | 68 (50,78) | 65 (51,85) | 68 (52,93) | -4 (-14,32) |  |  |
| Tertile 3 | 68 (64,89) | 68 (50,92) | 72 (58,96) | 3 (-6,20) |  |  |
| PQ interval-tertiles |  |  |  |  |  |  |
| Tertile 1 | 68 (51,82) | 67 (53,86) | 63 (52,81) | -2 (-16,15) | 0.988 |  |
| Tertile 2 | 66 (51,75) | 64 (52,77) | 65 (50,88) | -2 (-12,9) |  |  |
| Tertile 3 | 64 (53,75) | 63 (54,76) | 61 (54,74) | 0 (-18,22) |  |  |
| QRS amplitude-tertiles |  |  |  |  |  |  |
| Tertile 1 | 64 (51,78) | 62 (50,79) | 63 (51,85) | -3 (-18,13) | 0.592 |  |
| Tertile 2 | 67 (52,75) | 62 (53,74) | 62 (52,78) | -2 (-14,23) |  |  |
| Tertile 3 | 68 (52,79) | 71 (57,84) | 65 (55,78) | 0 (-16,11) |  |  |
| QRS width-tertiles |  |  |  |  |  |  |
| Tertile 1 | 64 (51,75) | 61 (48,74) | 60 (50,77) | -3 (-16,15) | 0.221 |  |
| Tertile 2 | 67 (51,79) | 61 (51,75) | 60 (51,76) | -4 (-16,7) |  |  |
| Tertile 3 | 68 (53,78) | 73 (60,84) | 70 (59,89) | 2 (-10,23) |  |  |
| QTc interval-tertiles |  |  |  |  |  |  |
| Tertile 1 | 59 (50,69) | 56 (51,71) | 60 (50,74) | 2 (-12,20) | 0.576 |  |
| Tertile 2 | 68 (55,76) | 64 (55,76) | 62 (53,89) | -3 (-16,15) |  |  |
| Tertile 3 | 72 (53,89) | 71 (60,87) | 68 (55,88) | -4 (-18,13) |  |  |
| Sokolow-Lyon index-tertiles |  |  |  |  |  |  |
| Tertile 1 | 65 (54,78) | 64 (54,83) | 65 (54,80) | -1 (-12,25) | 0.200 |  |
| Tertile 2 | 64 (51,73) | 60 (51,72) | 62 (51,75) | 1 (-14,13) |  |  |
| Tertile 3 | 68 (52,82) | 66 (55,78) | 61 (52,78) | -6 (-20,13) |  |  |
| Heart rate-tertiles |  |  |  |  |  |  |
| Tertile 1 | 64 (49,74) | 61 (47,76) | 60 (49,77) | 0 (-15,15) | 0.437 |  |
| Tertile 2 | 64 (54,77) | 60 (52,76) | 60 (53,77) | -4 (-15,12) |  |  |
| Tertile 3 | 69 (54,86) | 69 (61,90) | 73 (59,94) | 2 (-16,30) |  |  |

*LVEDV, left ventricular endsystolic volume; IQR, interquartile range; ms, milliseconds; mV, millivolt; bpm*

**Supplement table 4.** Changes in LVEDV over visits with respect to baseline ECG parameters (N = 181)

| **LVEDV** | **Baseline** | **6-weeks** | **26-weeks** | **% change** | **P-value** | **P-interaction** |
| --- | --- | --- | --- | --- | --- | --- |
|  | **Median (IQR)** | **Median (IQR)** | **Median (IQR)** | **Median (IQR)** |  |  |
| Cardiac axis deviation |  |  |  |  |  |  |
| Extreme axis | 152 (152-152) | 157 (157-157) | 179 (179-179) | 18 (18,18) | 0.345 | 0.176 |
| Left axis | 125 (104-141) | 130 (109-148) | 131 (115-154) | 6 (-6,25) |  |  |
| Normal axis | 124 (104-144) | 134 (109-161) | 132 (117-168) | 10 (-1,25) |  |  |
| Right axis | 114 (88-150) | 134 (126-147) | 148 (142-160) | 28 (7,61) |  |  |
| ST-elevation |  |  |  |  |  |  |
| No | 126 (104-139) | 132 (115-155) | 134 (117-153) | 10 (-2,27) | 0.339 | 0.497 |
| Yes | 124 (104-143) | 132 (106-152) | 132 (116-163) | 7 (-4,22) |  |  |
| ST-depression |  |  |  |  |  |  |
| No | 125 (107-141) | 132 (112-154) | 135 (118-158) | 9 (0,26) | 0.350 | 0.874 |
| Yes | 125 (98-141) | 132 (103-148) | 129 (112-163) | 5 (-7,22) |  |  |
| T-inversion |  |  |  |  |  |  |
| No | 126 (109-139) | 134 (110-150) | 130 (115-158) | 12 (-7,26) | 0.692 | 0.631 |
| Yes | 125 (104-145) | 131 (111-154) | 135 (117-159) | 7 (-2,25) |  |  |
| Ischemic change |  |  |  |  |  |  |
| No | 125 (104-141) | 132 (111-155) | 134 (117-162) | 8 (-4,25) | 0.695 | 0.404 |
| Yes | 115 (93-136) | 134 (108-136) | 123 (112-141) | 10 (1,20) |  |  |
| MI type |  |  |  |  |  |  |
| NSTEMI | 128 (104-143) | 133 (113-149) | 134 (108-152) | 9 (-11,23) | 0.910 | 0.463 |
| STEMI | 125 (104-141) | 133 (108-154) | 132 (117-160) | 8 (-3,25) |  |  |
| Q-wave |  |  |  |  |  |  |
| No | 124 (96-139) | 132 (110-146) | 128 (104-152) | 7 (-7,26) | 0.135 | 0.695 |
| Yes | 126 (112-148) | 134 (110-159) | 142 (120-163) | 9 (1,25) |  |  |
| Q-wave duration |  |  |  |  |  |  |
| <40 ms | 124 (111-130) | 128 (108-134) | 114 (107-141) | 8 (1,11) | 0.354 | 0.384 |
| ≥40 ms | 127 (112-148) | 138 (113-160) | 143 (122-168) | 10 (2,24) |  |  |
| P-wave amplitude (mv) |  |  |  |  |  |  |
| 0.05 | 132 (127-143) | 140 (131-148) | 143 (120-157) | 1 (-9,15) | 0.391 | 0.724 |
| 0.10 | 125 (104-141) | 132 (110-159) | 132 (115-167) | 9 (-2,25) |  |  |
| 0.15 | 119 (96-147) | 124 (99-147) | 132 (118-158) | 9 (-7,25) |  |  |
| ≥0.20 | 113 (84-131) | 133 (99-151) | 137 (98-148) | 15 (4,29) |  |  |
| Q-wave amplitude-tertiles |  |  |  |  |  |  |
| Tertile 1 | 127 (111,139) | 133 (125,149) | 128 (115,143) | 5 (1,12) | 0.092 |  |
| Tertile 2 | 128 (101,146) | 132 (108,160) | 146 (117,166) | 11 (2,25) |  |  |
| Tertile 3 | 123 (112,153) | 134 (106,166) | 150 (124,184) | 16 (4,30) |  |  |
| PQ interval-tertiles |  |  |  |  |  |  |
| Tertile 1 | 130 (104,146) | 133 (110,163) | 130 (114,152) | 6 (-7,20) | 0.264 |  |
| Tertile 2 | 124 (102,138) | 136 (107,151) | 141 (117,164) | 12 (3,25) |  |  |
| Tertile 3 | 125 (110,141) | 131 (114,148) | 128 (118,157) | 7 (-7,30) |  |  |
| QRS amplitude-tertiles |  |  |  |  |  |  |
| Tertile 1 | 122 (99,141) | 123 (100,148) | 135 (115,157) | 6 (-2,21) | 0.626 |  |
| Tertile 2 | 125 (102,143) | 127 (110,149) | 130 (117,152) | 10 (-4,29) |  |  |
| Tertile 3 | 128 (113,144) | 142 (126,163) | 140 (121,163) | 7 (-4,23) |  |  |
| QRS width-tertiles |  |  |  |  |  |  |
| Tertile 1 | 122 (98,141) | 122 (105,144) | 126 (112,149) | 9 (-5,27) | 0.570 |  |
| Tertile 2 | 126 (98,141) | 125 (101,152) | 127 (106,158) | 7 (-3,20) |  |  |
| Tertile 3 | 128 (108,147) | 142 (126,162) | 146 (128,166) | 9 (-4,25) |  |  |
| QTc interval-tertiles |  |  |  |  |  |  |
| Tertile 1 | 119 (97,134) | 126 (103,136) | 130 (104,148) | 9 (-3,28) | 0.707 |  |
| Tertile 2 | 126 (109,141) | 135 (110,154) | 137 (117,166) | 7 (0,23) |  |  |
| Tertile 3 | 134 (104,153) | 142 (115,165) | 142 (120,172) | 8 (-6,20) |  |  |
| Sokolow-Lyon index-tertiles |  |  |  |  |  |  |
| Tertile 1 | 125 (98,141) | 134 (110,157) | 138 (116,160) | 11 (2,22) | 0.225 |  |
| Tertile 2 | 119 (108,138) | 123 (106,150) | 130 (118,147) | 9 (-4,28) |  |  |
| Tertile 3 | 128 (104,147) | 135 (114,154) | 132 (114,166) | 3 (-7,25) |  |  |
| Heart rate-tertiles |  |  |  |  |  |  |
| Tertile 1 | 121 (97,141) | 129 (99,151) | 128 (106,154) | 8 (-5,25) | 0.882 |  |
| Tertile 2 | 124 (108,141) | 128 (112,153) | 136 (118,156) | 6 (0,25) |  |  |
| Tertile 3 | 129 (100,144) | 140 (117,154) | 138 (118,166) | 10 (-7,26) |  |  |

*LVEDV, left ventricular enddiastolic volume; IQR, interquartile range; ms, milliseconds; mV, millivolt; bpm*

**Supplement table 5.** Changes in Troponin T levels over visits with respect to baseline ECG parameters (N = 181)

| **Troponin T** | **Baseline** | **6-weeks** | **26-weeks** | **% change** | **P-value** | **P-interaction** |
| --- | --- | --- | --- | --- | --- | --- |
|  | **Median (IQR)** | **Median (IQR)** | **Median (IQR)** | **Median (IQR)** |  |  |
| Cardiac axis deviation |  |  |  |  |  |  |
| Extreme axis | 2310 (2310-2310) | 22 (22-22) | 11 (11-11) | -100 (-100,-100) | 0.809 | 0.115 |
| Left axis | 3444 (2310-5117) | 17 (12-21) | 10 (7-15) | -100 (-100,-100) |  |  |
| Normal axis | 2755 (1911-5060) | 16 (13-25) | 10 (7-14) | -100 (-100,-99) |  |  |
| Right axis | 5417 (4203-5500) | 26 (13-27) | 14 (12-18) | -100 (-100,-100) |  |  |
| ST-elevation |  |  |  |  |  |  |
| No | 2423 (1996-4551) | 16 (12-20) | 10 (7-13) | -100 (-100,-99) | 0.357 | 0.588 |
| Yes | 3596 (2466-5424) | 17 (13-24) | 11 (8-15) | -100 (-100,-100) |  |  |
| ST-depression |  |  |  |  |  |  |
| No | 2966 (2039-4816) | 17 (13-22) | 10 (7-14) | -100 (-100,-99) | 0.206 | 0.499 |
| Yes | 3951 (2423-5707) | 16 (12-22) | 11 (7-14) | -100 (-100,-100) |  |  |
| T-inversion |  |  |  |  |  |  |
| No | 3698 (2276-5422) | 17 (13-28) | 13 (8-15) | -100 (-100,-100) | 0.617 | 0.443 |
| Yes | 3073 (2073-5018) | 16 (12-21) | 10 (7-14) | -100 (-100,-100) |  |  |
| Ischemic change |  |  |  |  |  |  |
| No | 3115 (2099-5117) | 17 (12-22) | 10 (7-14) | -100 (-100,-100) | 0.404 | 0.792 |
| Yes | 3906 (2241-5021) | 17 (16-23) | 13 (10-14) | -100 (-100,-100) |  |  |
| MI type |  |  |  |  |  |  |
| NSTEMI | 2331 (1654-3444) | 17 (13-21) | 10 (8-14) | -100 (-100,-99) | 0.161 | 0.037 |
| STEMI | 3274 (2310-5294) | 17 (12-23) | 11 (7-14) | -100 (-100,-100) |  |  |
| Q-wave |  |  |  |  |  |  |
| No | 2802 (2000-4734) | 16 (13-21) | 10 (7-14) | -100 (-100,-99) | 0.677 | 0.675 |
| Yes | 3468 (2326-5360) | 17 (12-25) | 11 (8-15) | -100 (-100,-100) |  |  |
| Q-wave duration |  |  |  |  |  |  |
| <40 ms | 2775 (2016-4202) | 16 (12-20) | 9 (7-12) | -100 (-100,-99) | 0.653 | 0.140 |
| ≥40 ms | 4042 (2416-5467) | 17 (12-26) | 11 (8-15) | -100 (-100,-100) |  |  |
| P-wave amplitude |  |  |  |  |  |  |
| 0.05 | 2621 (1885-4585) | 17 (12-28) | 13 (6-16) | -99 (-100,-99) | 0.502 | 0.557 |
| 0.10 | 3185 (2189-5018) | 17 (12-22) | 10 (7-15) | -100 (-100,-100) |  |  |
| 0.15 | 4041 (2416-6400) | 18 (13-19) | 12 (7-14) | -100 (-100,-100) |  |  |
| ≥0.20 | 3869 (1954-5510) | 15 (13-26) | 10 (7-14) | -100 (-100,-100) |  |  |
| Q-wave amplitude-tertiles |  |  |  |  |  |  |
| Tertile 1 | 3066 (2039,5192) | 16 (12,24) | 8 (7,14) | -100 (-100,-100) | 0.711 |  |
| Tertile 2 | 3305 (2380,5319) | 16 (11,22) | 11 (8,16) | -100 (-100,-100) |  |  |
| Tertile 3 | 4326 (2450,6433) | 20 (17,27) | 12 (8,15) | -100 (-100,-100) |  |  |
| PQ interval-tertiles |  |  |  |  |  |  |
| Tertile 1 | 3244 (2241,5367) | 17 (13,22) | 10 (7,14) | -100 (-100,-100) | 0.577 |  |
| Tertile 2 | 3115 (2000,4647) | 16 (11,20) | 9 (7,14) | -100 (-100,-100) |  |  |
| Tertile 3 | 3152 (2387,5201) | 17 (13,26) | 12 (9,16) | -100 (-100,-99) |  |  |
| QRS amplitude-tertiles |  |  |  |  |  |  |
| Tertile 1 | 3464 (2030,5069) | 16 (12,22) | 10 (7,14) | -100 (-100,-100) | 0.584 |  |
| Tertile 2 | 3004 (2221,5364) | 17 (13,23) | 12 (7,15) | -100 (-100,-99) |  |  |
| Tertile 3 | 2795 (2241,5099) | 17 (12,22) | 11 (7,15) | -100 (-100,-100) |  |  |
| QRS width-tertiles |  |  |  |  |  |  |
| Tertile 1 | 3582 (1992,5330) | 17 (13,21) | 10 (7,14) | -100 (-100,-100) | 0.772 |  |
| Tertile 2 | 2591 (2215,4467) | 16 (11,21) | 10 (6,15) | -100 (-100,-99) |  |  |
| Tertile 3 | 3180 (2310,5294) | 17 (12,26) | 11 (8,16) | -100 (-100,-100) |  |  |
| QTc interval-tertiles |  |  |  |  |  |  |
| Tertile 1 | 3456 (2355,5294) | 17 (13,25) | 12 (8,14) | -100 (-100,-100) | 0.413 |  |
| Tertile 2 | 3244 (2099,4938) | 15 (12,19) | 9 (7,14) | -100 (-100,-100) |  |  |
| Tertile 3 | 2703 (2062,5380) | 19 (13,26) | 10 (7,15) | -100 (-100,-99) |  |  |
| Sokolow-Lyon index-tertiles |  |  |  |  |  |  |
| Tertile 1 | 4369 (2409,5507) | 17 (14,24) | 10 (8,14) | -100 (-100,-100) | 0.040 |  |
| Tertile 2 | 3039 (2000,4914) | 17 (12,26) | 10 (7,15) | -100 (-100,-99) |  |  |
| Tertile 3 | 2594 (2073,4056) | 16 (12,20) | 11 (7,15) | -100 (-100,-99) |  |  |
| Heart rate-tertiles |  |  |  |  |  |  |
| Tertile 1 | 3040 (2062,4701) | 16 (12,20) | 10 (7,14) | -100 (-100,-100) | 0.955 |  |
| Tertile 2 | 3070 (2000,5099) | 16 (13,23) | 11 (7,15) | -100 (-100,-99) |  |  |
| Tertile 3 | 3582 (2288,5707) | 18 (12,26) | 10 (7,15) | -100 (-100,-100) |  |  |

*IQR, interquartile range; ms, milliseconds; mV, millivolt;*
